# Gold(III)-Dithiocarbamato Peptidomimetics in the Forefront of the Targeted Anticancer Therapy: Preclinical Studies against Human Breast Neoplasia

**DOI:** 10.1371/journal.pone.0084248

**Published:** 2014-01-02

**Authors:** Chiara Nardon, Sara M. Schmitt, Huanjie Yang, Jian Zuo, Dolores Fregona, Q. Ping Dou

**Affiliations:** 1 Department of Chemical Sciences, University of Padova, Padova, Italy; 2 Molecular Therapeutics Program, Barbara Ann Karmanos Cancer Institute, Departments of Oncology, Pharmacology and Pathology, School of Medicine, Wayne State University, Detroit, Michigan, United States of America; 3 College of Chemistry and Chemical Engineering, Ocean University of China, Qingdao, Shandong, China; King Faisal Specialist Hospital & Research Center, Saudi Arabia

## Abstract

Since the serendipitous discovery of cisplatin, platinum-based drugs have become well-established antitumor agents, despite the fact that their clinical use is limited by many severe side-effects. In order to both improve the chemotherapeutic index and broaden the therapeutic spectrum of current drugs, our most recent anti-neoplastic agents, Au(III) complexes, were designed as carrier-mediated delivery systems exploiting peptide transporters, which are up-regulated in some cancers. Among all, we focused on two compounds and tested them on human MDA-MB-231 (resistant to cisplatin) breast cancer cell cultures and xenografts, discovering the proteasome as a major target both *in vitro* and *in vivo*. 53% inhibition of breast tumor growth in mice was observed after 27 days of treatment at 1.0 mg kg^−1^ d^−1^, compared to control. Remarkably, if only the most responsive mice are taken into account, 85% growth inhibition, with some animals showing tumor shrinkage, was observed after 13 days. These results led us to file an international patent, recognizing this class of gold(III) peptidomimetics as suitable candidates for entering phase I clinical trials.

## Introduction

According to the WHO, cancer is the second-leading cause of death worldwide after cardiovascular diseases. Cisplatin (*cis*-[Pt^II^Cl_2_(NH_3_)_2_]) was approved by the U.S. FDA in 1978 to treat genitourinary tumors and, currently, it is well-established as an effective drug for the treatment of testicular cancer and, in combination with other chemotherapeutic agents, for ovarian, cervical, brain, bladder, lung, and breast cancers [1]. Other platinum(II)-based drugs have been developed but very few are currently in clinical use, including carboplatin and oxaliplatin. At present, due to severe side-effects, such as neuro- and nephrotoxicity as well as tumor resistance [2], the design and development of alternative metal-based anticancer agents with better chemotherapeutic indices is still a great challenge in the field of medicinal chemistry [3]. In this context, we designed, synthesized and characterized several innovative metal (such as platinum, palladium, ruthenium, zinc and copper) complexes derived from different dithiocarbamato ligands. This class of ligands was chosen considering the chemo-protective effect of various sulfur-containing nucleophiles previously used clinically to lessen cisplatin nephrotoxicity. In fact, the renal toxicity rises from the strong and irreversible binding of platinum-based drugs to intracellular enzymes containing donor sulfur atoms, thus inactivating them and leading to renal failure. To date, our results demonstrate that combining the antitumor properties of some metal ions with a chemoprotective organic moiety is a promising strategy [4], [5].

More recently, we have been studying new gold(III) dithiocarbamato complexes and have observed remarkable biological activity both *in vitro* and *in vivo*. Notably, these compounds showed no cross-resistance to cisplatin, indicating a different mechanism of action [6]. In particular, we reported on the anti-proliferative activity of gold(III)-dithiocarbamato derivatives of amino acids toward a broad panel of tumor cell lines [7]–[10]. Moreover, negligible nephrotoxicity [11] and high anticancer activity was observed *in vivo* in nude mice bearing human breast or prostate cancer xenografts [7], [9].

**Figure 1:**
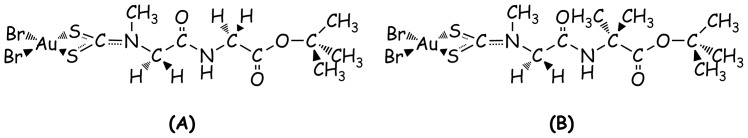
Chemical structures of investigated gold(III)-based compounds AuD6 (A) and AuD8 (B).

Based on these very encouraging results, we aimed to increase the bioavailability and tumor specificity of these novel compounds by exploiting peptide transporters. Peptide transporters are integral plasma membrane proteins that regulate the cellular uptake of di- and tripeptides and peptide-like molecules (*i.e.* peptidomimetics). To date, two peptide transporters (PEPT1 and PEPT2) have been discovered in mammals. They are localized chiefly in epithelial cells of the mammary glands, lungs, bile ducts, small intestine, kidneys and choroid plexus [12], [13], but are also present in other tissues (pancreas, liver, gastrointestinal tract) [14] and, interestingly, seem to be up-regulated in some cancer types such as osteosarcoma, bladder, prostate [15], gastric [15], [16], bile duct [17], pancreatic, fibrosarcoma, intestinal and renal [18]–[20]. They are able to transport most di- and tripeptides into the cell regardless of sequence, size, charge and hydrophobicity [21]. Thus, we synthesized and widely characterized a second generation of gold(III)-dithiocarbamato complexes with oligopeptides as ligands. Based on preliminary cell-line screenings [22], [23], we selected for further investigation the complexes AuD6 and AuD8 (([Au^III^Br_2_(dtc-Sar-AA-O(*t*-Bu))] with AA = Gly or Aib (α-aminoisobutyric acid), respectively)) whose ligands are di-peptide derivatives differing only in the substituents at the α-carbon atom at the *C*-terminus (Figure 1).

In the current study, we focused on the anticancer activity on the triple-negative human breast cancer (TNBC) cell line MDA-MB-231, as these cells are highly metastatic, invasive and resistant to cisplatin. In addition, we aimed to elucidate the mechanism of action, identifying the proteasome as a major target both *in vitro* and *in vivo*. The proteasome is an ATP-dependent protease and plays a key role in nuclear and cytoplasmic homeostasis by degrading a large range of proteins marked with poly-ubiquitin chains. Importantly, in spite of initial skepticism in the scientific community, proteasome inhibition has proven a winning strategy to treat malignancies. The ubiquitin-proteasome system (UPS) plays a major role in several intracellular processes such as cell cycle progression, apoptosis, DNA damage and repair, endocytosis, drug resistance, angiogenesis and cell differentiation [24], [25]. In tumor cells, protein homeostasis is unbalanced, rendering cancer cells more sensitive to inhibitors of the UPS than normal cells [26]. Among the proteasomal enzymes, the most studied is the 26S proteasome whose molecular weight is 2,500 kDa. 26S proteasome is formed by a barrel-shaped multicatalytic complex referred to as the 20S proteasome core particle (CP), capped at each end by a regulatory component termed the 19S complex [27]. The role of the 19S particles is to recognize ubiquitinated client proteins and mediate their entry to the 20S core regulating their unfolding and translocation into the proteolytic chamber. The CP (MW∼750 kDa) consists of 28 subunits, which are arranged in four axially stacked seven-membered rings, yielding a cylinder-like complex with a α_1–7_β_1–7_β_1–7_α_1–7_ stoichiometry. Eukaryotic 20S proteasomes contain only three proteolytically active β subunits per β ring (subunits β1, β2 and β5), whereas the remaining β subunits and α subunits are inactive. The β1, β2 and β5 subunits are responsible for three different catalytic activities: peptidyl glutamyl peptide hydrolyzing-like, trypsin-like and chymotrypsin-like (hydrolysis at the *C*-terminal side of acidic, basic and hydrophobic amino acid residues, respectively). In addition to chymotrypsin (CT)-like activity, BrAAP (branched chain amino acid-preferring activity) and SNAAP (small neutral amino acid-preferring activity) activities are ascribed to the β5 subunit as well [27], [28]. Encouragingly, our compounds exhibited potent proteasome inhibitory activity, associated with induction of cell death *via* apoptosis.

## Results

### Design of AuD6 and AuD8

Therapeutic effectiveness of drug candidates is limited by their abilities to traverse the plasma membrane. In order to overcome this, several carrier-mediated delivery strategies have been studied. As mentioned previously, complexes [Au^III^Br_2_(dtc-Sar-AA-O(*t*-Bu))] with AA = Gly (AuD6) or Aib (AuD8) (Figure 1A–B) were designed (following our previous studies on Au(III)-amino acid derivatives) as anticancer gold(III)-based peptidomimetics that might specifically exploit the transporters PEPT1 and PEPT2. We maintained the sarcosinedithiocarbamato moiety (dtc-Sar) [10] and selected the *C*-terminal amino acids to evaluate the effect of flexibility (Gly) and rigidity (α-aminoisobutyric acid, Aib) on the cytotoxic activity of the corresponding complexes. The choice of Aib, a natural noncoded amino acid, was not accidental. Aib shows peculiar conformational preferences [29], highly affecting the backbone structure of the final peptide, which is usually folded into either α helix or 3_10_ helical arrangement. Therefore, rigid Aib-containing molecules have the capability to alter cell membrane permeability and they exhibit antibiotic, antiviral and anticancer activities [30], [31]. Additionally, C^α^-tetrasubstituted amino acids are able to overcome resistance to enzymatic degradation. *C*-terminal esterification of our complexes was required as previous analogues containing a free carboxylic group proved inactive [32]. In order to provide stability to the final compounds, bulky *t*-butyl groups were chosen, thus decreasing the possibility of hydrolysis. Lastly, peptide transport studies often involve the sarcosine-containing dipeptide Gly-Sar as a reference substrate as it shows good affinity toward both PEPT1 and PEPT2 [33].

With reference to Figure S1A, the *t*-butyl-esterified dipeptides were prepared as hydrochlorides [22] by classical solution coupling strategies. The synthesis of the Au(III)-dithiocarbamato derivatives starting from the corresponding dipeptide ligands is shown in Figure S1B. Our gold-based compounds with two similar peptide ligands were prepared according to a general synthetic approach reported in the materials and methods section. Both compounds were characterized by elemental and thermogravimetric analyses, FT-IR and UV-Vis spectrophotometries, mono- and multidimensional NMR spectrometry. The crystal structure of AuD8 was also solved [22].

### Inhibition of MDA-MB-231 Cell Proliferation by AuD6 and AuD8 in Comparison with Cisplatin

MTT assay was used to assess the *in vitro* cytotoxicity of AuD6 and AuD8 against MDA-MB-231 cells after 24 or 72 h treatment. Although both complexes inhibited tumor cell growth in a concentration-dependent manner, AuD8 proved more potent than AuD6, with IC_50_ values ± SD of 6.5±0.6 and 17±1 µM, respectively. After 72 h treatment, the IC_50_ of AuD6 decreased to 13±1 µM. Notably, MDA-MB-231 cells were resistant to cisplatin under the same experimental conditions. In fact, after 24 h treatment the IC_50_ value was not reached using concentrations ranging from 25 to 100 µM in agreement with literature data [34]. At 100 µM cisplatin exhibited a growth-inhibitory effect of around 40% after 24 h treatment whereas after 72 h around 23% cell viability was observed in agreement with the known slower activity kinetics presented by the reference drug [35].

### Influence of the ROS Scavenger Trolox on the Anticancer Activity of Gold(III) Complexes

In order to elucidate if ROS species are produced intracellularly by our gold-based compounds, we co-treated MDA-MB-231 cells with both complexes and the hydrophilic form of vitamin E (Trolox), which is able to react with free radicals [36]. We carried out two independent experiments (Figure S2), and observed different results. First, we pretreated cells for 1 h with Trolox at 100 µM, followed by 24 h treatment with the compounds. We observed no changes in IC_50_ values for either compound, suggesting that cell growth inhibition is independent of ROS generation. In the second experiment, we co-treated cells with each of the two compounds and Trolox (100 µM) for 24 h. As expected, we observed higher antiproliferative activity in the presence of Trolox. In fact, Trolox alone can inhibit tumor cell growth by approximately 25% after 24 h treatment. Interestingly, Trolox reduced the IC_50_ of AuD6 approximately 3-fold, with very little effect on AuD8, and therefore, AuD6 displays a greater synergistic effect than AuD8, in combination with Trolox, in agreement with literature data [37].

### *In vitro* Inhibition of Proteasomal Activities by AuD6 and AuD8 on Purified 20S and Cell Extracts

Since first generation compounds were able to inhibit the CT-like (chymotrypsin-like) active site of the proteasome [7], [8], we hypothesized that these second generation complexes could target the tumor proteasome as well. To test this hypothesis, we investigated the effects of both compounds on the proteasomal CT-like, trypsin (T)-like and peptidyl-glutamyl-peptide-hydrolizing (PGPH)-like activities of MDA-MB-231 cell extracts. Both complexes were able to inhibit all the three proteasomal enzymatic activities (Table 1) in a concentration-dependent manner. However, AuD8 showed some selectivity for CT-like activity mediated by the β5 subunit [27], [28].

**Table 1:** *In vitro* inhibition of proteasome.

	Proteasomalactivity	AuD6	AuD8
***Purified 20S***	*Chymotrypsin-like*	0.42±0.03	0.29±0.07
	*Trypsin-like*	0.51±0.07	0.61±0.02
	*PGPH-like*	0.49±0.09	0.54±0.08
***Cell extract***	*Chymotrypsin-like*	3.3±0.4	2.9±0.6
	*Trypsin-like*	3.0±0.9	5.7±0.1
	*PGPH-like*	2.3±0.8	4.5±0.9

IC_50_ values (µM±SD) obtained for the three proteasomal activities on the purified 20S proteasome and on MDA-MB-231 cell extract after 2 h incubation.

To provide direct evidence for this selectivity, we incubated purified human 20S proteasome with each compound at different concentrations and the fluorogenic substrates specific for each activity, using DMSO as a control. We observed that AuD8 preferentially inhibited the CT-like activity of the purified 20S proteasome as well (Table 1). Remarkably, in terms of proteasome inhibition, both complexes proved to be about an order of magnitude more potent than the first generation compounds [7], [8]. Velcade® (bortezomib) was used as a positive control (IC_50_ = 2.2±0.6 nM for the CT-like pocket) (*see Discussion*).

### Treatment of Breast Cancer Cells with AuD6 and AuD8 Triggers Proteasome Inhibition and Apoptosis

In order to elucidate to what extent cellular proteasome is inhibited by our compounds we treated breast cancer cells with both complexes at different concentrations for 24 h, followed by measurement of proteasomal activities. AuD8 showed a greater degree of selectivity for the CT-like pocket than AuD6 in these cells. Notably, both compounds were also able to inhibit proteasomal PGPH-like (peptidyl-glutamyl-peptide-hydrolizing-like) activity (Figure S3).

**Figure 2:**
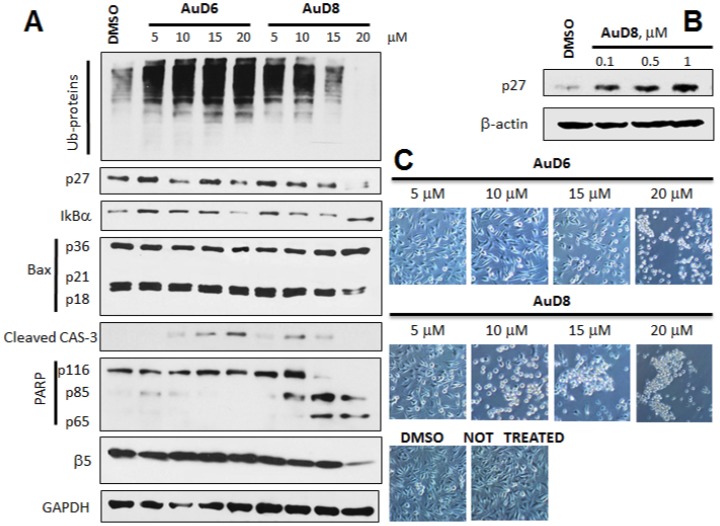
Western blot and morphological analysis (concentration-dependent study). **A**, Western blot analysis of breast cancer MDA-MB-231 cell extracts. Cells were treated with the complexes AuD6 and AuD8 at the indicated concentrations for 24 h. **B**, Western blot analysis of the p27 protein amount in MDA-MB-231 cell extract after treatment with the compound AuD8 at the indicated concentrations for 24 h. The solvent DMSO was used as a control while GAPDH as a loading control. **C**, Apoptotic morphological changes of MDA-MB-231 cells after treatment with AuD6 and AuD8 at the indicated concentrations for 24 h (phase contrast imaging, 100× magnification).

To gain insight into the mechanism of action of our compounds, we performed concentration- and time-dependent *in vitro* studies. MDA-MB-231 cells were treated with either (i) AuD6 or AuD8 for 24 h at different concentrations (5, 10, 15 and 20 µM) (Figure 2A–B) or (ii) each compound at 20 µM for 4, 8, 16 or 24 h (Figure S4A). Overall, we observed accumulation of ubiquitinated proteins, p27 and IκBα, caspase-3 activation, cleavage of poly(ADP-ribose) polymerase (PARP) and, interestingly, changes in levels of the proteasomal β5 subunit after treatment with AuD8. Treatment with AuD8 also resulted in enhanced levels of p36/Bax and consequent decrease or complete disappearance of p21/Bax and p18/Bax fragments. In fact, it has been reported that Bax protein (p21/Bax) could be hydrolyzed by calpain, thus yielding a p18/Bax fragment. This cleavage is associated with cell death commitment and leads to formation of the p36/Bax homodimer. Gao *et al*. reported that p18 and p36/Bax are more potent in disrupting mitochondrial integrity than full-length Bax, followed by release of mitochondrial cytochrome c, activation of caspase-3 and cleavage of poly(ADP-ribose) polymerase (PARP) [38]. Importantly, progressive decrease in full-length caspase-3 in Figure S4A is in agreement with the increase in levels of cleaved caspase-3 (active form) in Figure 2A. Additionally, Figures 2 and S4 highlight the affinity of AuD8 for the proteasomal β5 subunit, as at higher concentrations and longer times the band intensity decreases, indicating that upon treatment with AuD8, some modifications occur in this protein subunit. Accordingly, such an interaction may account for the accumulation of ubiquitinated proteins, the proteasomal target proapoptotic proteins p27 and IκB-α that leads to tumor growth inhibition/apoptosis.

The complex AuD6 was less potent than AuD8 in agreement with its higher IC_50_ value, reported above. Indeed, we observed bands as intense as the control for some proteins while weaker effects were observed on the apoptotic phenomena (*i.e.,* PARP cleavage and caspase-3 activation) following treatment with higher concentrations or for longer time periods (Figures 2A and S4A).

**Figure 3:**
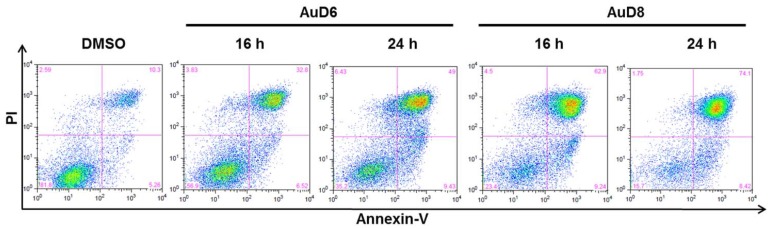
Annexin–V FITC/PI assay. MDA-MB-231 cells were treated with the complexes AuD6 and AuD8 (20 µM) for 16 and 24 h. Then, cells were labeled with Annexin–V FITC and PI and analyzed by flow cytometry in order to evaluate the percentage of apoptotic cells. Apoptotic cells at early stage occur in the lower right quadrant while apoptotic cells at late stage set in the up-right part. The percentage in the lower left quadrant is due to viable cells whereas the upper left part to non-apoptotic cell death.

To further confirm that cell death takes place *via* apoptosis, we carried out an Annexin V/Propidium Iodide (PI) assay. MDA-MB-231 cells were treated with AuD6 or AuD8 for 16 or 24 h at 20 µM. Cells were then harvested and stained with Annexin-V FITC and PI prior to flow cytometry. Remarkably, the amount of cells undergoing non-apoptotic cell death was comparable to the solvent control (Figure 3). After 16 h treatment with AuD8 the majority of cell death (62.9%) occurred by apoptosis (late stage) and this percentage further increased after 24 h (74.1%). Although both compounds showed similar percentages of cells in early stage apoptosis (around 7–9%), AuD6 proved less potent than AuD8 as it induced late stage apoptosis in only 49% of cells after 24 h.

### *In vivo* Experiments

**Figure 4:**
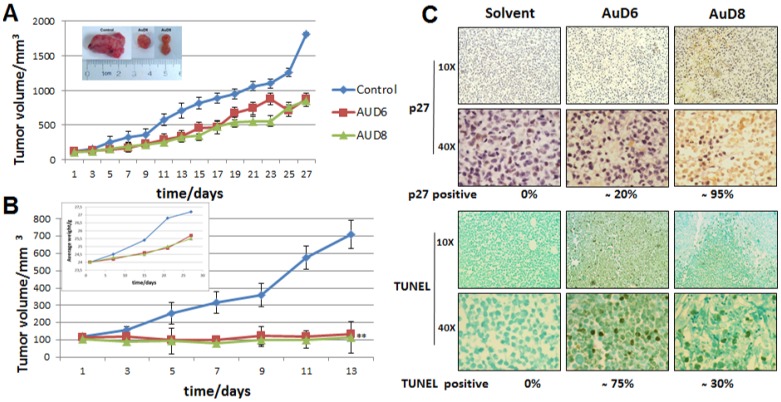
Antitumor activity *in vivo* on MDA-MB-231 xenografts. Female nude mice bearing MDA-MB-231 tumors were treated with either vehicle (control) or the compounds AuD6 and AuD8 at 1 mg kg^−1^ d^−1^. **A,** Inhibition of xenograft growth by both complexes. Tumor volumes were measured every other day using a caliper. Points represent the mean ± SD (bars) of seven mice per group. The insert depicts representative tumors from each treatment group; * = p<0.05. **B**, If only the most responsive mice are considered, the xenograft growth inhibition is greater. The insert shows average weights of mice over time; ** = p<0.01. **C**, Immunohistochemical p27 and TUNEL staining of tumor samples indicates proteasome inhibition and apoptosis as a result of both compounds. Stronger p27 staining is observed following AuD8 treatment, and more TUNEL positive cells are observed following AuD6 treatment. Brown colored cells are considered positive.

We investigated the anticancer activity of both compounds *in vivo* in female athymic nude mice bearing human breast cancer MDA-MB-231 xenografts. During treatment mice did not display signs of fatigue, weight loss (See the Insert, Figure 4B) or anorexia. Mice were randomly divided into three groups of seven, and treated daily with either the control or AuD6 or AuD8 (1.0 mg kg^−1^ d^−1^). At day 13, three mice per group were sacrificed to determine if anti-tumor effects could be observed after only such a short treatment period, and the remaining four mice per group were sacrificed at day 27 or when control tumors reached approximately 1,800 mm^3^. AuD8 showed slightly higher anticancer activity than AuD6 (Figure 4A); AuD8-treated tumors grew to 849±79 mm^3^, corresponding to 53% inhibition of tumor growth compared to control (p<0.05; Figure 4A, and Insert). When only the most responsive mice are taken into account (40%), 85% inhibition was observed after 13 days (p<0.01; Figure 4B), with some mice exhibiting tumor shrinkage.

**Figure 5:**
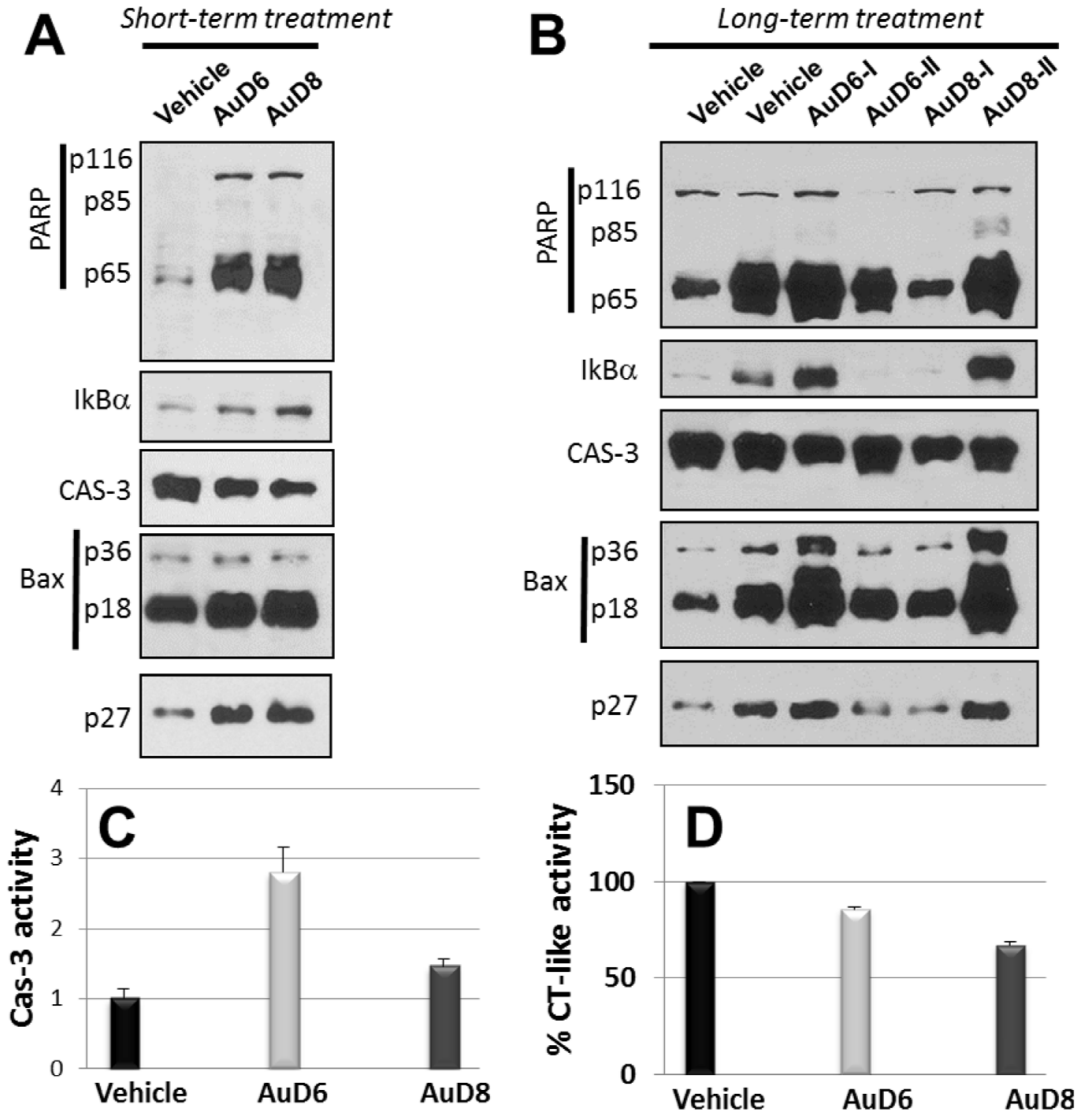
Western blot *in vivo* analysis. Female nude mice bearing MDA-MB-231 tumors were treated with either vehicle (control) or the compounds AuD6 and AuD8 at 1 mg kg^−1^ d^−1^. Tumors were collected and the corresponding tissues prepared for Western blot analysis after either 13-day treatment (**A**) or 27-day treatment (**B**) [I and II denote distinct experiments]. Tissues were also prepared for the assays of caspase-3 activity (**C**) and of proteasomal CT-like activity (**D**) after 27 days of treatment.

The immunohistochemical data in mouse xenografts indicate that AuD8 treatment resulted in a significant (∼95%) increase in levels of p27 expression compared to control (Figure 4C). A moderate increase (∼20%) in p27 was observed following AuD6 treatment. These results are similar to those seen *in vitro*, with AuD8 being a more potent proteasome inhibitor than AuD6. Western blot analysis confirmed the proteasome-inhibitory activity of these gold-based compounds. AuD8 is slightly more potent toward IκB-α and Bax accumulation than AuD6 (Figure 5A and B). Additionally, AuD8 was a more potent proteasome inhibitor than AuD6, resulting in 33% and 14% inhibition of the CT-like pocket, respectively (Figure 5D).

Proteasome inhibition *in vivo* was accompanied by apoptosis induction in tumors treated with these gold compounds. AuD6 treatment resulted in a higher level of TUNEL (Terminal deoxynucleotidyl transferase dUTP nick end labeling assay) positivity compared to AuD8 treatment (∼75% vs. ∼30%; Figure 4C) while AuD8 treatment gave higher levels of p27 staining (95% vs. 20%; Figure 4C). Consistently, AuD6 was a more potent activator of caspase-3 than AuD8 (Figure 5A–C). We noticed the variation in the replicates (Figure 4B), representing various individual mouse background. The treatment with both compounds also resulted in inhibition of the proteasomal CT-like activity (Figure 5D). Taken together, our data could suggest that proteasome inhibition may not be the only major mechanism responsible for apoptotic cell death mediated by these gold compounds, and that these gold-based compounds showed potent proteasome-inhibitory and apoptosis-inducing activities *in vivo*, with very little-no toxicity, evident by no decrease in mouse body weight (Figure 4B, Insert).

## Discussion

Recently, coordination compounds have received considerable attention due to their distinctive properties compared to organic molecules. The choice of the metal center, its oxidation state and the types of ligand (*O*-, *N*-, *S*-donors, monodentate or chelating ones etc.) determines the traits, including the coordination geometry and stereochemistry, of the final compound, resulting in unique kinetic and thermodynamic properties. For instance, modification of the oxidation state of the metal center radically changes the structural and chemical properties and the resulting biological activity of a complex [4], [5], [24]. Therefore, the peculiar chemistry of metal-based drugs leads to unexpected pharmacological profiles due to novel mechanisms of action at the molecular level.

Here, we have shown that the gold(III) dithiocarbamato-peptide derivatives AuD6 and AuD8 were able to inhibit MDA-MB-231 tumor growth both *in vitro* (IC_50_ = 6.5±0.6 and 17±1 µM, respectively) and *in vivo* at a low dose of 1.0 mg kg^−1^ per day. The MDA-MB-231 cell line is a TNBC, usually associated with poor prognosis and lack of benefit from both hormonal therapies (based on estrogen and progesterone receptor antagonists) and monoclonal antibody therapy targeting the human epidermal growth factor receptor 2 [39]. Thus, MDA-MB-231 tumor develops aggressively and frequently leads to early gastrointestinal metastases [40]. In this study, we observed 53% inhibition of xenograft growth compared to control treatment after 27 days (Figure 4A). Six mice (three for each compound) showed 85% inhibition and, in some cases, tumor shrinkage after 13 days of treatment (Figure 4B). Notably, mice appeared healthy and very active throughout the treatment. Overall, the toxicological and anticancer activity results point out that the design of our compounds proved very successful in both stabilizing the heavy metal center and in avoiding the aspecific reactivity of the compound occurring in healthy tissues that could give rise to systemic toxicity [5]. As described above, the ligands should carry the corresponding complexes into the tumor cells. In this context, we are currently investigating the transport into the cell to determine whether uptake occurs by PEPT transporters, as expected.

Being coordination compounds of a redox-active noble metal, our complexes were also investigated for their ability to produce ROS species in the cellular milieu after treatment. The enhancement of the anticancer activity of these compounds by co-treatment with Trolox suggests that cell death commitment is triggered by apoptotic mechanisms independent of free radicals formed *in situ* (*see below*).

It is known that cancerous cells are characterized by unbalanced cell homeostasis and are more sensitive than normal ones to apoptosis-inducing agents [24], [25]. In this context, bortezomib (Velcade®), a boron-containing inorganic pharmaceutical, is the first-in-class proteasome inhibitor, approved by the FDA in 2003 for the treatment of some hematologic malignancies including multiple myeloma [41]. Bortezomib forms a reversible covalent complex with the proteasomal β5 subunit, thus blocking proteolysis and therefore leading to cell death [42]. The drug mainly targets the proteasomal β5 active site (β5> β1>> β2) but its binding to the three subunits essentially occurs in the same way [43]. Indeed, due to its Lewis acidity, boron binds to the oxygen atom on the side chain of the *N*-terminal threonine (Thr1O^γ^) of each subunit, thus yielding a proteasomal Thr1O^γ^-drug tetrahedral complex. Our gold compounds are also potent proteasome inhibitors and, although AuD6 and AuD8 closely resemble cisplatin from the structural point of view (*i.e*., square planar geometry with a d^8^ electronic configuration), the latter is not a proteasome inhibitor, possibly explaining the resistance of MDA-MB-231 cells to cisplatin (IC_50_>100 µM after 24 h) *in vitro*. Like bortezomib, our compounds are electrophilic species. In particular, both types of inhibitors are coordination compounds [24] and consist of an electrophilic trap, the semimetal element boron for the former and the metal center Au(III) for the latter. In its compounds, boron may form an extra reversible bond in order to complete the octet whereas the transition metal gold(III) forms four covalent bonds in a square-planar geometry *via* an associative mechanism. Similar to boron in bortezomib, the metal center Au(III) could bind to the proteasomal active sites, likely irreversibly, but it may also be involved in a redox process that would lead to the formation of gold(I)-containing species or metallic gold by oxidation of some residue of the multicatalytic protease such as the nucleophilic threonine.

*In vitro* AuD8 showed a degree of selectivity toward the β5 subunit (CT-like). As mentioned above, in addition to CT-like activity, BrAAP and SNAAP activities are ascribed to the β5 subunit as well [27], [28]. Overall, these properties could account for the selectivity observed for AuD8 toward the β5 subunit in the cell-free system. In fact, considering the peptide portion of our inhibitors, AuD8 is slightly more hydrophobic and more branched than AuD6. AuD6 showed no selectivity toward a specific proteolytic subunit as comparable IC_50_ values were observed for all three activities in both the purified proteasome and MDA-MB-231 cell extracts (Table 1). Importantly, it has been shown that targeting the CT-like activity is associated with apoptosis induction in cancer cells [44]. However, recently, co-targeting the PGPH-like and/or T-like (trypsin-like) sites has been suggested as a new strategy in treating malignancies because it increases the cytotoxicity of proteasome inhibitors [45], [46]. Based on this finding, Goldberg *et al*. reported that when one proteolytic activity is inhibited, the two others are increasingly important in protein breakdown and the subsequent inactivation of either of the remaining catalytic sites has a greater effect on decreasing proteolysis than when one site alone is inhibited. Thus, the complete or near complete inhibition of all three sites seems to be required to induce cell death [45]. Accordingly, we hypothesize that a cascade effect on the different proteasomal subunits may take place after interaction of the multicatalytic protease with our metal complexes.

In this work, we observed morphological changes such as shrinkage and characteristic apoptotic blebbing with increasing compound concentration or over time (Figures 2C and S4B, respectively). In kinetic experiments, cells treated with both compounds detached and rounded up after only 4 h and these apoptotic features were significantly enhanced after 24 h. In concentration-dependent studies, cells treated with AuD6 appeared detached and clustered only at 20 µM whereas upon treatment with AuD8 at 10 µM several shrunken cells were observed in good agreement with the calculated IC_50_ values. In this context, annexin V-FITC staining pointed out cell death takes place *via* apoptosis rather than necrosis. Apoptotic cell death *via* proteasome inhibition, both *in vitro* (Figures 2A, S4A) and *in vivo* (Figure 5), has been associated with a variety of down-stream effects, most notably accumulation of ubiquitinated proteins and proteasomal target proteins like p27, Bax and IκBα. The observed accumulation of IκBα results in sustained inhibition of the cell growth promoting protein NF-κB, which contributes to cell death [47]. Concerning p27 accumulation, we showed that AuD8 was able to induce it also at very low concentration (below 1 µM, Figure 2B). Additionally, increased p27 staining and TUNEL positivity were detected in xenografts treated with both compounds, validating the proteasome-inhibitory and apoptosis-inducing activities *in vivo* as well (Figures 4C–5C–D). However, AuD8 treatment resulted in greater p27 staining than AuD6 whereas AuD6 treatment caused stronger TUNEL staining, highlighting that apoptosis induction may not be solely due to the proteasome-inhibitory abilities. Consequently, multiple targets may be responsible for cancer cell death triggered by these gold compounds.

In conclusion, we identified the proteasome as a major *in vivo* and *in vitro* target of our compounds recording encouraging data that allowed us to file an international patent for their use in cancer chemotherapy (just extended in several Countries worldwide) [48]. Nevertheless, although the proteasome has been recognized as one of the potential targets of our gold compounds, we have also been focusing on the interaction of the complexes with other biomolecules in order to discover other possible cellular targets, but also to elucidate their pharmacokinetics. Furthermore, through the advanced preclinical studies required for entering phase I clinical trials, we are now gaining insight into gene modification upon treatment as well.

## Materials and Methods

### Statement

All animal work was carried out at the Barbara Ann Karmanos Cancer Institute- Dept. of Oncology (Wayne State University, MI) in strict accordance with the recommendations in the Guide for the Care and Use of Laboratory Animals of the National Institutes of Health. The protocol was approved by the Institutional Animal Care and Use Committee of Wayne State University (Permit Number: A 04-11-10). All efforts were made to minimize suffering.

### Materials

The chemicals for the synthesis of the gold(III) dithiocarbamato derivatives AuD6 and AuD8 and their wide physico-chemical characterization have been described elsewhere [22]. Fluorogenic peptide substrates Suc-LLVY-AMC, Z-ARR-AMC, and Z-LLE-AMC (for the proteasomal chymotrypsin-like, trypsin-like and PGPH-like activities, respectively) and Ac-DEVD-AMC (for caspase-3 like activity) were purchased from Calbiochem (San Diego, CA). Purified human 20S proteasome was from Boston Biochem (Boston, MA). EZ-RUN Pre-stained Rec protein ladder was purchased from Fisher Scientific (Pittsburgh, PA), 40% bis-acrylamide 29∶1 solution and TEMED for electrophoresis were from Bio-Rad (Hercules, CA). DMEM/F12 cell growth medium, sodium pyruvate, HEPES, penicillin, and streptomycin were purchased from Invitrogen (Carlsbad, CA) while standard fetal bovine serum was from Aleken Biologicals (Nash, TX). Rabbit monoclonal antibody against cleaved caspase-3 (active form, 5A1, Asp175) was purchased from Cell Signaling Technology (Danvers, MA). Mouse monoclonal antibodies against ubiquitin (P4D1), p27 (F-8), Bax (B-9), IκB-α (H-4), GAPDH (9B3), rabbit polyclonal antibody against caspase-3 (H-277), and secondary antibodies were from Santa Cruz Biotechnology, Inc. (Santa Cruz, CA). Rabbit polyclonal antibody against human proteasomal β5 subunit and human poly(ADP-ribose) polymerase (PARP) were purchased from Enzo Life Sciences (Farmingdale, NY). FITC Annexin V apoptosis detection kit I was purchased from BD Biosciences (San Jose, CA). Sodium azide, NaCl, Tris base, sterile DMSO, 3-(4,5-Dimethyl-2-thiazolyl)-2,5-diphenyl-2H-tetrazolium bromide (MTT), *cis*-diammineplatinum(II) dichloride (hereinafter, cisplatin), Tween-20, cremophor, ethanol, and other chemicals) were from Sigma-Aldrich (St. Louis, MO).

All chemicals were of high grade pureness and used as purchased without any further purification.

### Synthesis of the Complexes

An aqueous solution (1.5 mL) of NaOH (0.27 mmol) was added to a water solution (3 mL) of HCl•Sar-AA-O*t*Bu (0.27 mmol). Then, CS_2_ (15 µL, 0.27 mmol) was added to the mixture. When the pH dropped from 9 to 6 (after about 3 hours), the solution was slowly added with continuous stirring to an aqueous solution (2 mL) of dihydrate K[AuBr_4_] (0.27 mmol), leading to the immediate precipitation of an orange solid that was filtered off, washed with water and lastly, dried *in vacuo* over P_4_O_10_, with a final yield of around 80%.

**AuD6.**C_10_H_17_AuBr_2_N_2_O_3_S_2_ (MW. 634.16) Elemental analysis %: (calculated) found C (18.94) 19.20, H (2.70) 2.88, N (4.42) 4.42, S (10.11) 10.45.

**AuD8.**C_12_H_21_AuBr_2_N_2_O_3_S_2_ (MW. 662.21) Elemental analysis %: (calculated) found C (21.76) 21.55, H (3.20) 3.09, N (4.23) 3.93, S (9.68) 9.32.

### Cell Culture and Cell Proliferation Assay

Human breast cancer MDA-MB-231 cells were obtained from American Type Culture Collection (Manassas, VA) and grown in DMEM/F12 supplemented with 10% fetal bovine serum, 1 mM sodium pyruvate, 10 mM HEPES, 100 U/mL penicillin and 100 µg/mL streptomycin. Cells were grown in a humidified incubator with 5% CO_2_ at 37°C. Cells were seeded in octuplicate in 96-well plates and grown to 70%–80% confluence, followed by treatment with DMSO (control) or each compound (dissolved in DMSO) in fresh medium at the indicated concentrations. After 24 or 72 hours incubation at 37°C, inhibition of cell proliferation was measured by MTT assay, as previously described [49]. The cytotoxicity of the compounds was quantified as the percentage of cells surviving relative to untreated cells. Four MTT tests for each compound were performed in order to evaluate the corresponding IC_50_ values.

For comparison purposes, cells were plated in quadruplicate in 96-well plates and grown to 70%–80% confluence, followed by treatment with cisplatin (dissolved in 0.9% NaCl_(aq)_) in fresh medium at 3 different concentrations.

In order to investigate whether cell death is affected by free radicals produced *in situ* by our compounds, cells were seeded in quadruplicate and treated with both complexes and Trolox. We first pretreated cells for 1 h with Trolox at 100 µM and after changing medium, treated cells with compounds and incubated for an additional 24 h. Alternatively, we co-treated cells for 24 h with both compounds and Trolox (100 µM), followed by MTT assay.

### Proteasome Activity Assay in Purified 20S Proteasome and MDA-MB-231 whole-Cell Extract

Purified human 20S proteasome (0.5 nM) was incubated in triplicate with three different proteasomal fluorogenic substrates (20 µM) and each compound (dissolved in DMSO) at various concentrations ranging from 0.1 to 10 µM (or an equivalent volume of solvent DMSO as a control) in 100 µL of assay buffer [20 mM Tris-HCl (pH 7.5)]. After 1 hour and 2 hours incubation at 37°C, inhibition of each proteasomal activity was measured [50], recording the fluorescence of hydrolyzed AMC groups using a Wallac Victor^3^ 1420 multilabel plate reader (PerkinElmer, Waltham, MA) with an excitation wavelength of 355 nm, an emission wavelength of 460 nm, and measurement time of 0.1 s.

Likewise, MDA-MB-231 whole-cell extract (10 µg/well) was incubated in quadruplicate with each complex and each fluorogenic substrate in 100 µL of assay buffer [20 mM Tris-HCl (pH 7.5)]. Fluorescence data were acquired after 1 hour and 2 hours incubation at 37°C. The whole-cell extract was prepared as described elsewhere [44].

### Proteasome Activity Assay in Intact Breast Cancer MDA-MB-231 Cells

MDA-MB-231 breast cancer cells were plated in quadruplicate and grown to around 75% confluence, followed by treatment with both complexes at different concentrations for 24 h at 37°C (DMSO as a control). The exhausted medium was then removed and fresh medium and proteasomal fluorogenic substrates (20 µM) were added to each well. Plates were incubated for 24 h at 37°C prior to recording fluorescence at 460 nm.

Only two proteasomal activities (CT-like and PGPH-like) were measured since our complexes showed some selectivity in studies on the purified enzyme. Besides, the arginine-rich fluorogenic substrate (positively charged) for the T-like activity was not used owing to its poor capability in cell-membrane crossing.

### Cellular Morphology and Western Blot Analyses

A Zeiss (Thornwood, NY) Axiovert 25 microscope was used for cellular morphology microscopic imaging with phase contrast.

MDA-MB-231 cells were grown to around 75% confluence, treated with gold-based complexes or solvent as a control, harvested, and lysed. Cell lysates (30 µg/lane) were separated by SDS-PAGE and transferred to a nitrocellulose membrane. After blocking of non-specific binding (5% non-fat dry milk in Tris-Buffered Saline with Tween 20-TBST), membranes were incubated overnight at 4°C with primary antibodies, followed by washing with TBST and incubation for 2 h with secondary antibodies. Bands of interest were visualized using HyGLO Chemiluminescent HRP Antibody detection reagent, HyBlot Chemiluminescence films (Denville Scientific Inc.; Metuchen, NJ) and a HOPE Micro-MAX film processor.

### Apoptosis Assay

Apoptosis indexes were measured using the Annexin-V fluorescein isothiocyanate (FITC) apoptosis detection kit I from by BD Pharmingen. MDA-MB-231 cells were grown to around 75% confluence, treated with gold-based complexes (20 µM) or solvent as a control for 16 or 24 h, harvested by means of a scraper and centrifugation. Cell pellets were washed twice with ice cold PBS and cells were then resuspended in 1× binding buffer at a concentration of 1•10^6^ cells/mL. 100 µL of the suspension was then transferred to a 5 mL flow cytometry tube and 5 µL FITC Annexin V and 5 µL PI for double stained samples (either 5 µL of FITC Annexin V or 5 µL of PI for controls) were added. Mixtures were gently vortexed and incubated for 15 min at RT in the dark followed by addition of 400 µL 1× binding buffer to each tube. Then, analysis by flow cytometry occurred within 1 h at the Microscopy, Imaging and Cytometry Resources (MICR) Core at the Karmanos Cancer Institute, Wayne State University. Data (Figure 3) are presented as density plots of Annexin-V (x-axis) and propidium iodide (PI, y-axis) stainings. Unstained cells, cells stained with either PI or FITC Annexin-V only, and untreated cells stained with both PI and Annexin-V were used to set up compensation and quadrants. The excitation wavelength was 488 nm and the detection wavelengths were 530±15 and 620±21 nm for Annexin V and PI, respectively. Cells staining negative for both markers were considered viable since Annexin V and PI are both cell membrane impermeable while cells staining positive for both markers were considered in late apoptosis. Cells staining positive for Annexin only were considered in early apoptosis whereas cells staining positive for PI only were considered dead by a necrotic pathway. Percentages of viable, apoptotic, and necrotic cells are reported in the corner of each quadrant (Figure 3).

### Human Breast Tumor Xenograft Experiments

Seven-week-old female athymic nude mice were purchased from Harlan Laboratories (Indianapolis, USA) and housed in accordance with protocols approved by the Institutional Laboratory Animal Care and Use Committee of Wayne State University. Human breast cancer MDA-MB-231 cells suspended in 0.1 mL of serum-free DMEM/F12 cell growth medium were inoculated subcutaneously (s.c.) into the right flank of each mouse. When tumors reached a volume of around 115 mm^3^, the mice were randomly allocated into three groups (seven mice per group) and treated five days a week by s.c. injection of either vehicle [1∶1∶1 v/v PBS, DMSO, Cremophore/ethanol (1∶4)] or medium containing 1.0 mg/kg of AuD6 or AuD8. Tumor length (L) and width (W) were measured every other day using a caliper, and tumor volumes were evaluated according to the standard formula (π ⋅ L ⋅ W^2^)/6. Mouse weights were monitored weekly. Four mice per group were sacrificed after 27 days of treatment, or when tumors reached ∼1,800 mm^3^, and the remaining three mice per group were sacrificed after 13 days to observe any early effects of treatments. The tumors were collected and weighed. Tumor tissues were used to measure proteasome inhibition and caspase-3 activation by enzymatic activity assays and by Western blot analysis.

### Immunohistochemistry

IHC was performed using a previously reported protocol [51]. Briefly, tumor samples were paraffin-embedded by the Pathology Core at Karmanos Cancer Institute (Detroit, MI, USA). Samples were then cut and stained for p27 or TUNEL by the BioBank at William Beaumont Hospital (Royal Oak, MI, USA). Anti-p27 (VP-P951) was from Vector Laboratories (Burlingame, CA, USA) and was used at a 1∶30 dilution followed by detection by DAKO. Samples were counterstained with DAB/Hematoxylin. TUNEL staining was performed using anti-digoxigenin and peroxidase substrate, followed by counterstaining with methyl green. In both cases, brown colored cells were considered positive.

### Statistical Analysis

Analysis of the in vivo tumor growth curves was performed using the one-sided ANOVA test in Excel®. p<0.05 was considered significant.

## Supporting Information

Figure S1 Synthetic scheme **(A)** for the preparation of dipeptides HCl•H-Sar-AA-O(t-Bu) (AA = Gly (AuD6), Aib (AuD8)): (1) ZOSu, TEA, CH_3_CN/H_2_O, r.t., 4 d; (2) isobutene, H_2_SO_4_ (cat.), CH_2_Cl_2_, −70°C/r.t., 7 d; (3) 30% Pd-C, H_2_, CH_2_Cl_2_; (4) NMM, isobutylchloroformiate, THF/CHCl_3_, −15°C/r.t., o.n.; (5) 10% Pd-C, H_2_, MeOH; (6) HCl/Et_2_O. Template reaction **(B)** exploited for the preparation of the complexes [Au^III^Br_2_(dtc-Sar-AA-O(*t*-Bu))] with AA = Gly (AuD6) or Aib (AuD8).
(TIF)

Figure S2 **ROS evaluation.** Growth inhibition curves obtained after treatment of MDA-MB-231 tumor cells for 24 h at different concentrations of AuD6 **(A)** or AuD8 **(B)** either in the absence or in the presence (1 h pretreatment or 24 h co-treatment) of the ROS scavenger TROLOX.
(TIF)

Figure S3 **Inhibition of proteasome after treatment.** Inhibition of the proteasomal CT-like and PGPH-like activities in MDA-MB-231 cells after 24 h treatment with AuD6 **(A)** and AuD8 **(B)**.
(TIF)

Figure S4 **Western blot and morphological analysis (time-dependent study). A,** Western blot analysis of breast cancer MDA-MB-231 cell extracts. Cells were treated with the complexes AuD6 and AuD8 (20 µM) over the indicated times. The solvent DMSO was used as a control while GAPDH as a loading control. **B**, Apoptotic morphological changes of MDA-MB-231 cells after treatment with AuD6 and AuD8 at 20 µM for the indicated times (phase contrast imaging, 100× magnification).
(TIF)
