# Supplementary figures and images for: Gold(III)-Dithiocarbamato Peptidomimetics in the Forefront of the Targeted Anticancer Therapy: Preclinical Studies against Human Breast Neoplasia
Source: PLoS One. 2014 Jan 2;9(1):e84248. doi: 10.1371/journal.pone.0084248 (PMC3879379; doi:10.1371/journal.pone.0084248)

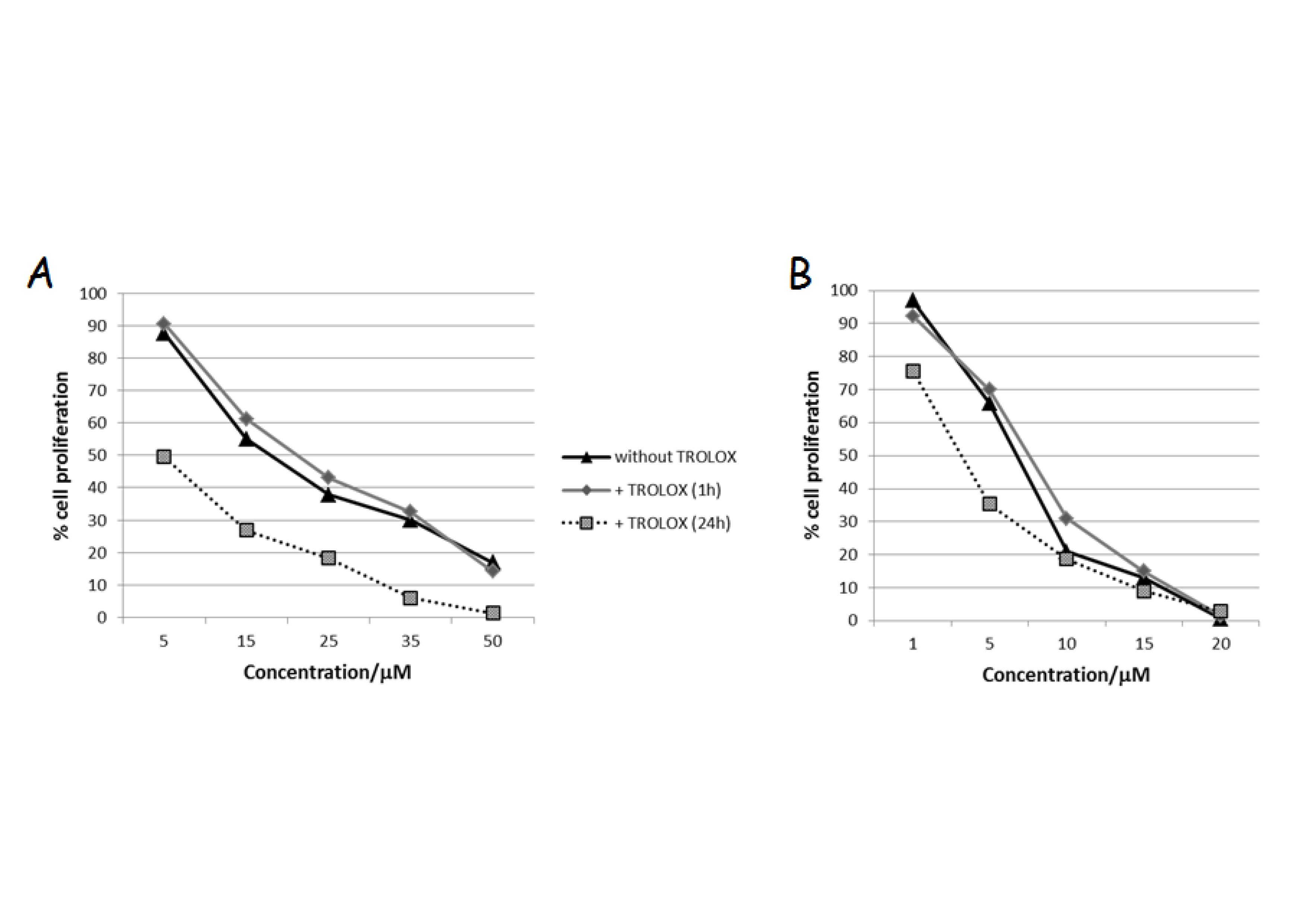

Supplement: Figure S2 — ROS evaluation. Growth inhibition curves obtained after treatment of MDA-MB-231 tumor cells for 24 h at different concentrations of AuD6 (A) or AuD8 (B) either in the absence or in the presence (1 h pretreatment or 24 h co-treatment) of the ROS scavenger TROLOX. (TIF) [file pone.0084248.s002.tif]

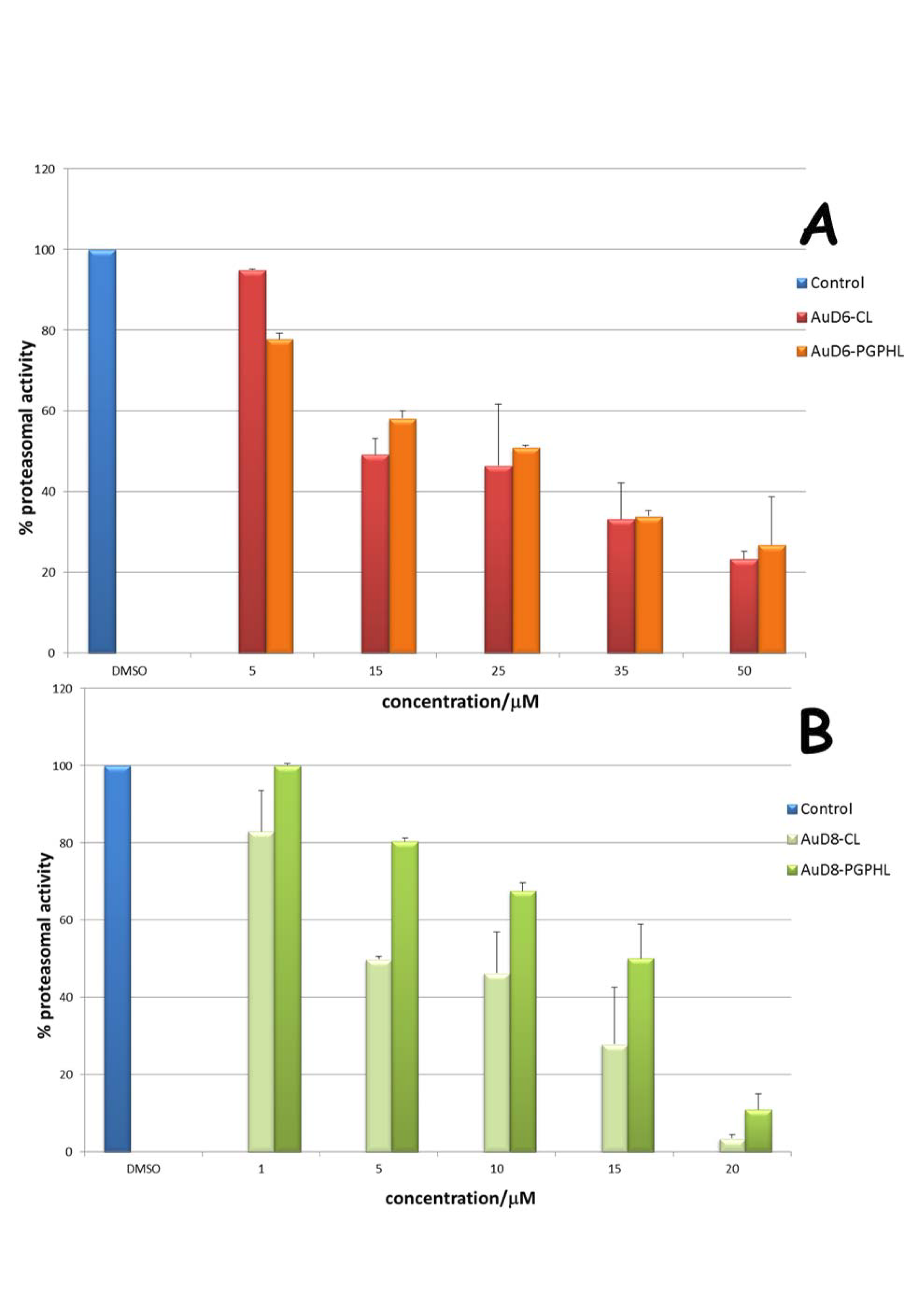

Supplement: Figure S3 — Inhibition of proteasome after treatment. Inhibition of the proteasomal CT-like and PGPH-like activities in MDA-MB-231 cells after 24 h treatment with AuD6 (A) and AuD8 (B). (TIF) [file pone.0084248.s003.tif]

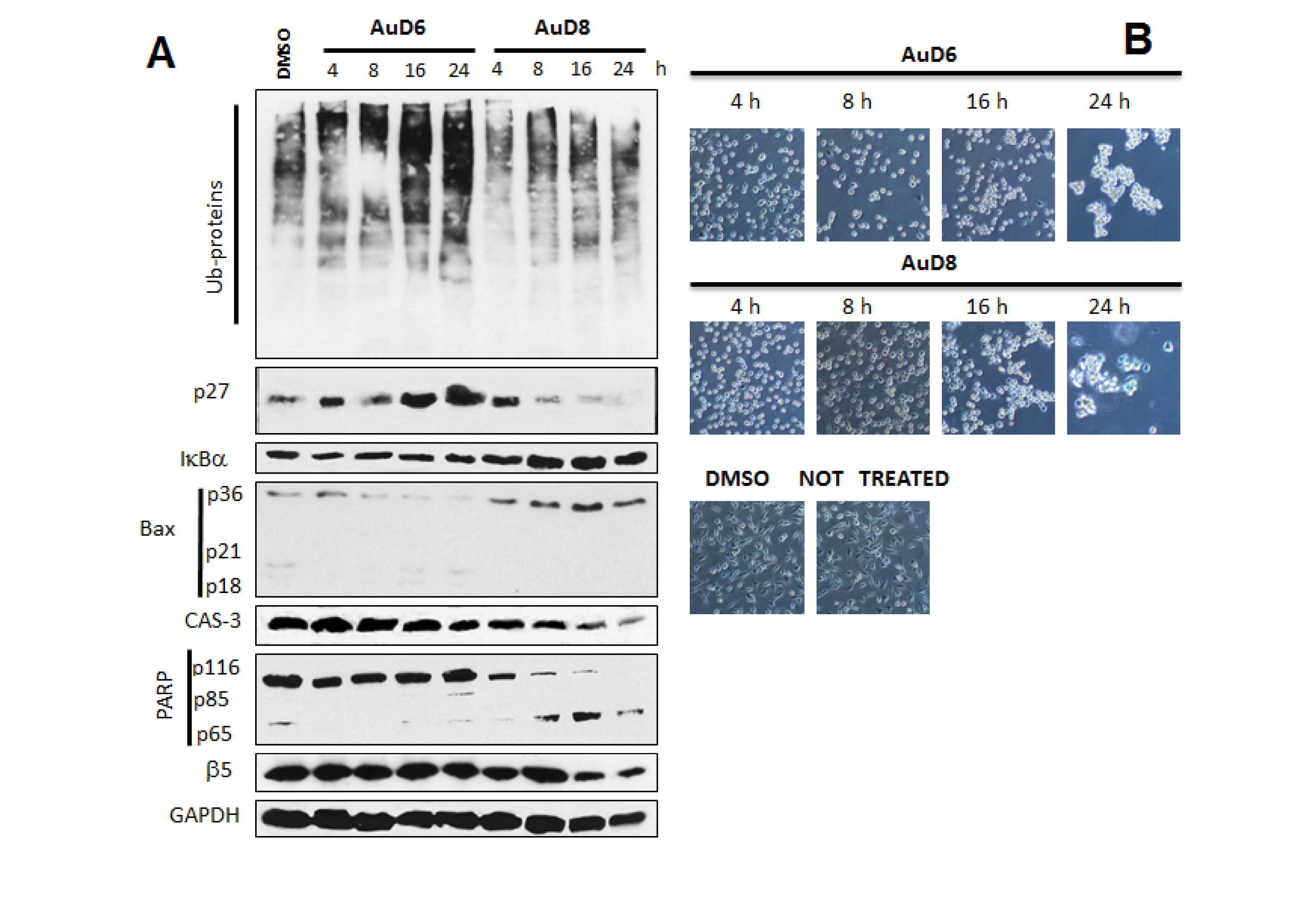

Supplement: Figure S4 — Western blot and morphological analysis (time-dependent study). A, Western blot analysis of breast cancer MDA-MB-231 cell extracts. Cells were treated with the complexes AuD6 and AuD8 (20 µM) over the indicated times. The solvent DMSO was used as a control while GAPDH as a loading control. B, Apoptotic morphological changes of MDA-MB-231 cells after treatment with AuD6 and AuD8 at 20 µM for the indicated times (phase contrast imaging, 100× magnification). (TIF) [file pone.0084248.s004.tif]
